# TLR7 activation by miR-21 promotes renal fibrosis by activating the pro-inflammatory signaling pathway in tubule epithelial cells

**DOI:** 10.1186/s12964-023-01234-w

**Published:** 2023-08-18

**Authors:** Jeongwon Kim, Sugyeong Ha, Minjung Son, Doyeon Kim, Mi-Jeong Kim, Bumseok Kim, Donghwan Kim, Hae Young Chung, Ki Wung Chung

**Affiliations:** 1 Department of Pharmacy and Research Institute for Drug Development, College of Pharmacy, Pusan National University, Busan, 46241 Republic of Korea; 2 Biosafety Research Institute and Laboratory of Veterinary Pathology, College of Veterinary Medicine, Jeonbuk National University, Iksan, 54596 Korea; 3 Functional Food Materials Research Group, Korea Food Research Institute, Wanju-Gun, 55365 Republic of Korea

**Keywords:** TLR7, Renal fibrosis, Inflammation, miR-21

## Abstract

**Background:**

Toll-like receptor 7 (TLR7) is an endosomal TLR activated by single-stranded RNA, including endogenous microRNAs. Although TLR7 is known to promote inflammatory responses in pathophysiological conditions, its role in renal fibrosis has not been investigated. Here, we aim to investigate the inflammatory roles of TLR7 in kidney inflammation and fibrosis.

**Methods:**

TLR7 knockout mice (*Tlr7* ^−/−^) subjected to AD-induced kidney injury were utilized to examine the role of TLR7 in kidney fibrosis. To elucidate the role of TLR7 in renal epithelial cells, NRK52E rat renal tubule epithelial cells were employed.

**Results:**

Under fibrotic conditions induced by an adenine diet (AD), TLR7 was significantly increased in damaged tubule epithelial cells, where macrophages were highly infiltrated. TLR7 deficiency protected against AD-induced tubular damage, inflammation, and renal fibrosis. Under in vitro conditions, TLR7 activation increased NF-κB activity and induced chemokine expression, whereas TLR7 inhibition effectively blocked NF-κB activation. Furthermore, among the known TLR7 endogenous ligands, miR-21 was significantly upregulated in the tubular epithelial regions. In NRK52E cells, miR-21 treatment induced pro-inflammatory responses, which could be blocked by a TLR7 inhibitor. When the TLR7 inhibitor, M5049, was administered to the AD-induced renal fibrosis model, TLR7 inhibition significantly attenuated AD-induced renal inflammation and fibrosis.

**Conclusions:**

Overall, activation of TLR7 by endogenous miR-21 in renal epithelial cells contributes to inflammatory responses in a renal fibrosis model, suggesting a possible therapeutic target for the treatment of renal fibrosis.

Video Abstract

**Supplementary Information:**

The online version contains supplementary material available at 10.1186/s12964-023-01234-w.

## Background

Chronic kidney disease (CKD) is a growing public health burden worldwide, affecting 8–16% of the global population and serving as the 10^th^ leading cause of death worldwide [1]. Despite intensive clinical and experimental research, the number of therapeutic targets for preventing CKD progression is limited. A final common pathway in CKD is kidney fibrosis, which involves the formation of internal scar tissue that leads to devastating effects [2]. In response to different types of tissue damage, initial responses, such as inflammation, naturally proceed to the wound healing process. Normal tissue repair can lead to a progressive fibrotic response if the tissue injury is severe, or if the wound healing process itself becomes unregulated. Excessive accumulation of fibrous connective tissue (components of the extracellular, such as collagens and fibronectin) in and around inflamed tissues leads to permanent scarring and tissue dysfunction, ultimately leading to organ failure [3]. Fibrosis is commonly associated with unresolved inflammation [4]. In the absence of resolution, inflammation involving infiltrating immune cells, in conjunction with intrinsic renal cells, fosters a pro-fibrotic environment [5].

Toll-like receptors (TLRs) are key regulators of the inflammatory response, and are associated with several cellular processes activated during kidney injury [6]. Among the TLR family, TLR7 is an endosomal innate immune sensor that can detect single-stranded ribonucleic acid [7]. Constitutive expression of TLR7 is predominant in dendritic cells and B cells compared to other immune cells, and low levels of TLR7 expression have been observed in non-immune cells, such as hepatocytes, epithelial cells, and keratinocytes [8]. Under inflammatory conditions, TLR7 expression is inducible in cells expressing low to undetectable basal TLR7 levels, including immune and non-immune cells [8]. TLR7-mediated pro-inflammatory responses are important for antiviral immune response. Altered TLR7 expression and the related pathways have been implicated in several autoimmune diseases, indicating a key role for this receptor in modulating inflammation.

MicroRNAs (miRNAs) are small non-coding RNAs with a length of approximately 19–25 nucleotides that critically modulate post-transcriptional regulation by targeting the complementary regions of coding and non-coding transcripts. miRNAs are involved in the regulation of various biological processes, including the modulation of the innate immune response, adaptive immune response, and inflammation [9]. Changes in miRNAs are implicated in pathological conditions, and owing to their role in many human diseases, these molecules are important mediators that must be studied in depth in medical research [10]. Recent studies have suggested various physiological roles of miRNAs, in addition to their direct regulatory role. In fact, several recent studies suggested that miRNAs are physiological ligands for TLRs that activate the inflammatory signaling cascade of the immune response [11, 12]. Under pathological conditions, an increase in specific miRNAs and their interaction with TLRs are important during disease development.

Although increased TLR7 has been implicated in several autoimmune and inflammatory diseases, its role in kidney disease development has not been extensively investigated. To our knowledge, this is the first study to demonstrate the involvement of TLR7 in kidney inflammation and fibrosis. By performing various biochemical experiments, including in situ hybridization, TLR7 was found to be highly expressed in the damaged tubule epithelial cells of fibrotic kidneys. Further in vivo experiments with TLR7 deficient mice revealed a significant contribution of TLR7 to the development of kidney fibrosis. Under in vitro conditions, TLR7 activation in renal epithelial cells directly increased pro-inflammatory NF-κB signaling in tubule epithelial cells. Further, we suggested that miR-21 can act as an endogenous TLR7 activator. Finally, a TLR7 inhibitor significantly reduced renal inflammation and fibrosis in animal models. Overall, our results suggest that activation of TLR7 by endogenous miR-21 contributes to inflammatory responses in a renal fibrosis model, suggesting a possible therapeutic target for the treatment of renal fibrosis.

## Methods and materials

### Animal studies

All animal experiments were performed in accordance with the guidelines for animal experimentation issued by the PNU and were approved by the Institutional Animal Care Committee of Pusan National University (PNU, IACUC approval No. PNU-2021–2937). Wild-type (WT) C57BL/6 J mice were obtained from Hyochang Science (Daegu, Korea). TLR7 knockout (*Tlr7* ^−/−^) mice were provided by Dr. Shizou Akira (Osaka University, Suita, Japan). Genotyping was performed using a KAPA Mouse Genotyping Kit (#KK7302, Sigma). To determine the role of TLR7 in chronic kidney disease, male WT mice (8 weeks old, *n* = 5–6) and male TLR7 knock out (KO) mice (8 weeks old, *n* = 5–6) were fed a 0.25% adenine diet (AD) for 3 weeks. To determine the effect of the TLR7 inhibitor on kidney injury, a folic acid (FA, dissolved in 0.3 M NaHCO3)-induced kidney fibrosis model was utilized. The TLR7 inhibitor, M5049 (0.3 mg/kg) (dissolved in 0.1 M Na-citrate buffer pH 3.0), was orally administered daily for 2 weeks. For all studies, mice were maintained at 23 ± 2° C with relative humidity of 60 ± 5% under 12 h light/dark cycles. At the end of each study, serum was collected for biochemical analysis, and the harvested kidneys were fixed in neutral buffered formalin for histochemical examination or frozen at − 80 °C for further biochemical experiments.

### Cell culture experiments

NRK52E rat renal tubular epithelial cells were purchased from ATCC (CRL-1571) and grown in Dulbecco's modified Eagle’s medium (DMEM) supplemented with 10% fetal bovine serum (FBS) and 1% penicillin. All cells were incubated at 5% CO_2_ and 37 °C in a water saturated atmosphere. To determine whether inflammation increases TLR7 expression, the cells were treated with 10 µg/mL lipopolysaccharide (LPS, L2630, Sigma). To investigate the role of TLR7 activation, the cells were treated with 50 µg/mL imiquimod (R837, HY-B0180, MedChemExpress) for 1 h. MiR-21-5p miRNA mimic and control mimic, synthesized by Genolution (Seoul, Korea), were used to elucidate the role of miR-21-5p in the activation of TLR7. Cells were transfected with the miR-21-5p mimic or control mimic using Lipofectamine 3000 (Thermo Fisher Scientific, Waltham, MA, USA), following the manufacturer’s instructions. For transfection, the cells were seeded in 60 mm cell culture dishes, grown overnight at approximately 70% confluence, and then transfected with 20 μM (final concentration) miR-21-5p mimic or control mimic. To confirm whether miR-21-5p activates inflammatory signaling through TLR7 activation, a TLR7 antagonist (M5049, HY-134581, MedChemExpress) was administered as a pretreatment 30 min prior to miR-21-5p transfection. For cell culture experiments, at least three different experiments were performed to ensure reproducibility.

### Serum biochemical measurements

Serum samples were collected via centrifugation at 3,000 rpm for 20 min at 4 °C. Blood urea nitrogen (BUN) levels were measured using a commercial assay kit from Shinyang Diagnostics (SICDIA L-BUN, 1,120,171, Seoul, Korea), according to the manufacturer's instructions.

### Protein extraction and western blot analysis

All tubes, solutions, and centrifuges were maintained at 4 °C. ProEXTM CETi protein extract solution (Translab, Daejeon, Korea) containing a protease inhibitor cocktail and a phosphate inhibitor was used to extract the total protein lysates from tissues. RIPA buffer (#9806, Cell Signaling Technology, MA, USA) containing a protease inhibitor cocktail was used to extract the total protein from cells. All buffers were used in accordance with the manufacturer’s instructions. Western blot analysis was performed to detect proteins from the kidneys or cells, as previously described [13]. The antibodies are listed in Supplementary Table 1.

### Isolation of total RNA, microRNA, and qRT-PCR

Total RNA was isolated using TRIzol reagent (Invitrogen, Carlsbad, CA, USA), following the manufacturer’s instructions. Total RNA (2,000 ng) was reverse-transcribed into cDNA using the SuPrimeScript RT-PCR Premix (SR-4100, GenDEPOT, Katy, TX, USA). The synthesized cDNA was stored at 4 °C and the miRNAs were extracted using Hybrid-RTM miRNA (Geneall, Seoul, Korea), according to the manufacturer’s instructions. The isolated miRNAs were synthesized into cDNA using M-MLV Reverse Transcriptase (E-3121, Bioneer, Daejeon, Korea) after linking the poly (A) tail using the EZ™ Poly(A) Tailing Kit (EZ041S, Enzynomics, Daejeon, Korea). All synthetic procedures were performed according to the manufacturer's instructions. Quantitative real-time PCR (qPCR) was performed using SYBR green dye (Applied Biosystems) and the CFX Connect System (Bio-Rad). qPCR data were analyzed and normalized using the 2-ΔΔCt method. The mRNA primers were designed using Primer3Plus, while the miRNA primers were designed using miRBase. The primer sequences are listed in Supplementary Table 2.

### Histological analysis

The kidneys were fixed in 10% neutral formalin, and paraffin-embedded sections were stained with hematoxylin and eosin (H&E) to examine the histological changes. Sirius Red staining was performed using a VitroView™ Picro-Sirius Red Stain Kit (VB-3017, Vitrovivo, Rockville, MD, USA) to visualize collagen I and III. Immunohistochemical staining (IHC) was performed using a commercially available kit from Vector Laboratories (Burlingame, CA, USA), according to the manufacturer's instructions, to measure the protein expression levels in paraffin-embedded kidney sections. Briefly, paraffin-embedded sections were incubated with the indicated primary antibodies (1:100–1:200 dilution; Supplementary Table 1). After blocking avidin and biotin using the Avidin/Biotin Blocking Kit (SP-2001, Vector Lab), the sections were incubated with the biotinylated secondary antibody and visualized using diaminobenzidine (DAB) substrates. The sections were counterstained with Mayer's hematoxylin (30,002, Muto Pure Chemicals, Japan). Images were obtained using a microscope (LS30; Leam Solution, Seoul, Korea).

### In situ hybridization

In situ hybridization (ISH) was performed using freshly prepared formalin-fixed, paraffin-embedded tissue samples. Single detection of mRNA was performed using the RNAscope 2.5 HD Assay-Red (322,500, Biotechne, Minneapolis, MN, USA). Dual detection of mRNA was performed using the RNAscope 2.5 HD Duplex Detection Kit (322,436, BioTechne). miRNA detection was performed using the miRNAscope HD Reagent Kit (324,500, Bio-techne). All experiments were performed according to the manufacturer’s instructions. The following probes were used for ISH: Mm-Tlr7 cat#415,411, Mm-Emr1 cat# 317,969-C2, Mm-Col1a1 cat# 319,379, Mm-Ccl2 cat# 311,791, and miR-21-5p cat# 729,041-S1. Images were captured using a microscope (LS30). In order to assess the specificity of the probe, we incorporated both positive and negative control probes provided in the detection kit.

### Determination of the transcriptional activity

Luciferase assays were performed to determine the transcriptional activity of NF-κB in NRK52E cells. After transfection with the NF-κB promoter-LUC plasmid, the luciferase activities were measured using the One-Glo Luciferase Assay System (Promega, Madison, WI, USA) and a luminescence plate reader (Berthold Technologies GmbH & Co., Germany).

### Immunofluorescence

The cells were fixed in 4% formaldehyde for 10 min, washed three times with ice-cold PBS, and exposed to 0.25% Triton-X 100 in PBS for 10 min for permeabilization. After blocking with 1% BSA/0.1% Tween 20 in PBS at room temperature for 30 min, the cells were incubated overnight with anti-TLR7 or anti-NF-κB antibodies diluted in blocking buffer at 4 °C. The cells were washed with PBS, incubated with a secondary antibody for 1 h in the dark, and counterstained with Hoechst 33,258 in PBS for 1 min. Images were acquired using a fluorescence microscope (LS30).

### Quantification and statistical analysis

To analyze the data obtained from cell culture experiments, non-parametric tests were utilized. Specifically, Mann Whitney tests was used to determine the differences between two groups. To analyze the data obtained from animal experiments, Student’s *t*-test was used to determine the differences between two groups. Statistical significance was set at *P* < 0.05. The analyses were conducted using Prism 5 software (GraphPad) and the images were assessed using ImageJ software.

## Results

### Changes in the TLRs in the AD-induced renal fibrosis model

**Fig. 1:**
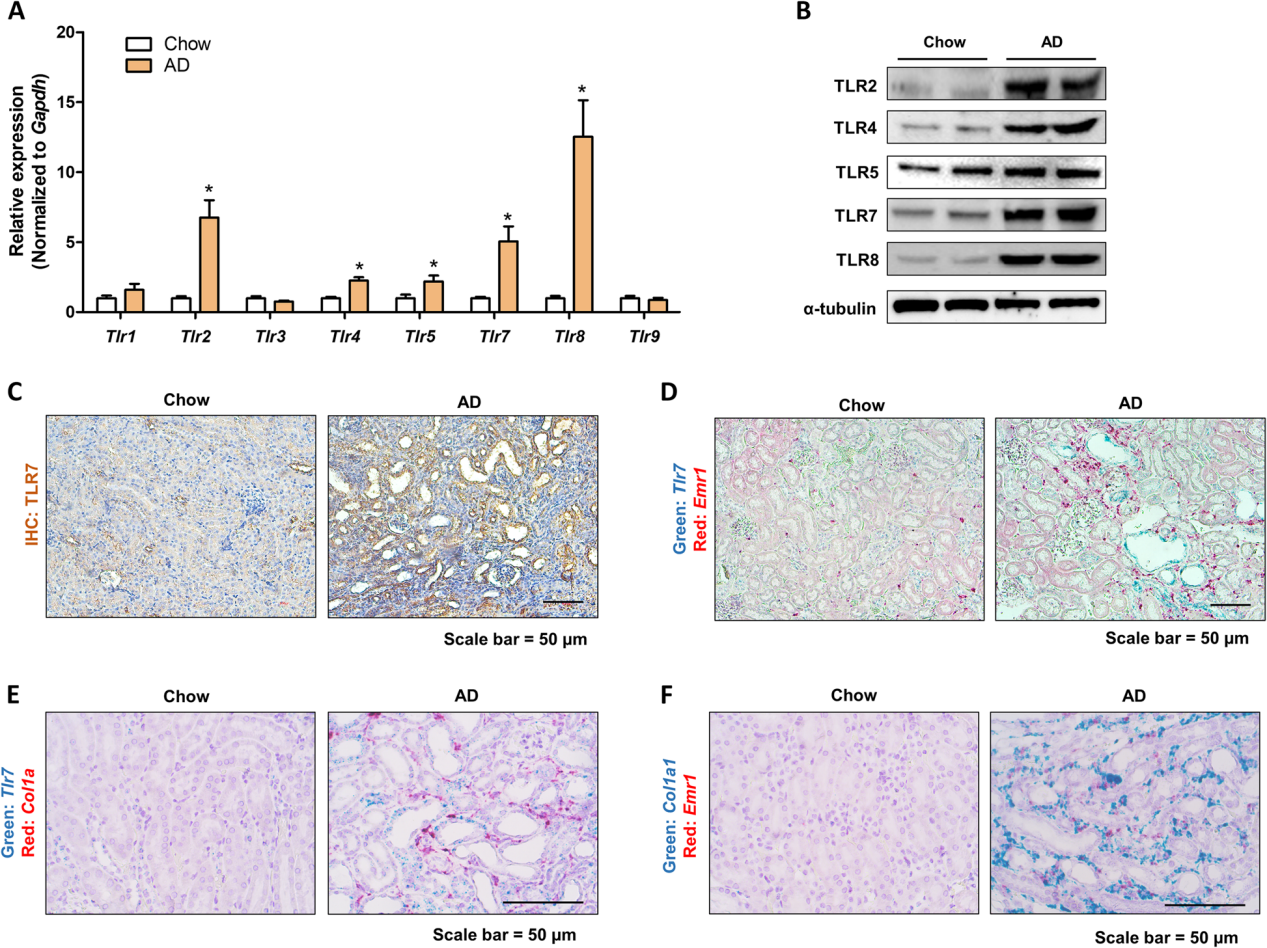
Changes in the TLR family gene and protein levels in adenine diet-induced renal fibrosis model. **A** Relative mRNA levels of the TLR family genes (Tlr1, Tlr2, Tlr3, Tlr4, Tlr5, Tlr7, Tlr8, and Tlr9) in the kidneys of mice fed normal chow diet or adenine diet for 3 weeks. *p < 0.05 versus normal chow diet-fed mice. **B** TLR2, TLR4, TLR5, TLR7, and TLR8 protein levels in kidneys were detected using western blotting. α-tubulin was used as a loading control. **C** Representative immunohistochemical (IHC) images of TLR7 expression in kidneys of normal chow- or adenine diet-fed mice. Scale bar = 50 μm. **D** Representative in situ hybridization (ISH) images of the Tlr7 (Green) and Emr1 (Red) genes in the kidney of mice fed chow diet or adenine diet. Scale bar = 50 μm. **E** Representative ISH images of the Tlr7 (Green) and Col1a1 (Red) genes in the kidney of mice fed chow diet or adenine diet. Scale bar = 50 μm. **F** Representative ISH images of the Col1a1 (Green) and Emr1 (Red) genes in the kidney of mice fed chow diet or adenine diet. Scale bar = 50 μm

To identify the role of TLRs in a renal fibrosis model, we confirmed the expression levels of TLR family genes and proteins in a 0.25% AD-induced renal fibrosis mouse model. qPCR revealed significant increases in *Tlr2, Tlr4, Tlr5, Tlr7,* and *Tlr*8 expression in the AD model (Fig. 1A). Further, we confirmed whether increased gene expression was associated with protein expression. The protein levels were also found to increase in the AD model (Fig. 1B). As the role of TLR7 activation in a kidney fibrosis model has not been investigated, IHC was employed to check the expression of TLR7 to determine its role in renal fibrosis. IHC revealed increased expression of TLR7 in the AD model, mainly in damaged tubule epithelial cells (Fig. 1C). The expression of *Tlr7* was further confirmed using ISH analysis. In accordance with the IHC results, increased *Tlr7* mRNA expression was detected in the damaged tubules (Fig. 1D). Interestingly, *Tlr7* expression was highly associated with macrophage infiltration in the kidneys. The macrophage marker, *Emr1*, was only detected near Tlr7-expressed tubule cells (Fig. 1D). Furthermore, *Col1a1* mRNA expression was also highly detected near *Tlr7*-expressing tubule cells (Fig. 1E), and *Col1a1* expression was also colocalizing with *Emr1*-positive macrophages, implicating its role in inflammation and fibrosis (Fig. 1F). Thus, TLR7 is increased in the damaged tubules of fibrotic kidneys and is associated with increased inflammation.

### TLR7 deficiency reduces renal damage and fibrosis in the AD model

**Fig. 2:**
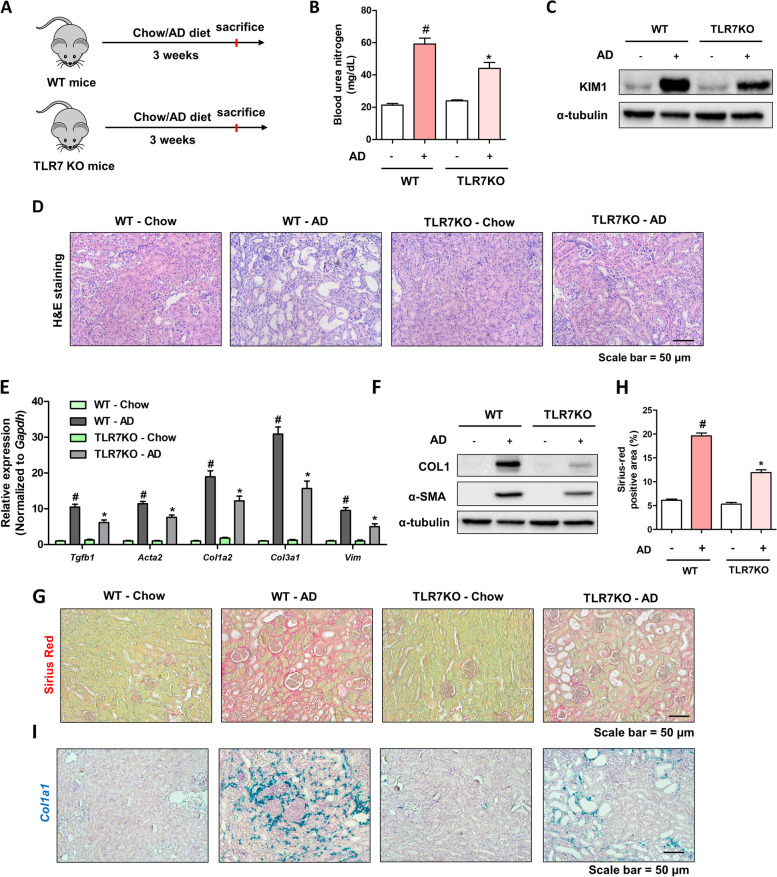
TLR7 deficiency reduces renal damage and fibrosis in the adenine diet model. **A** Study design for the administration of a chow diet or adenine diet to WT and TLR7 knock out (KO) mice. **B** Serum BUN levels of WT and TLR7 KO mice fed a chow diet or adenine diet. #*p* < 0.05 versus chow diet-fed WT mice. *p < 0.05 versus adenine diet-fed WT mice. **C** KIM-1 protein levels were detected in the kidneys of WT and TLR7 KO mice fed a chow diet or adenine diet for 3 weeks via western blotting. α-tubulin was used as a loading control. **D** Representative H&E staining images of the kidneys of WT and TLR7 KO mice fed a chow diet or adenine diet for 3 weeks. Scale bar = 50 μm. **E** Relative mRNA levels of fibrosis-associated genes (Tgfb, Acta2, Col1a2, Col3a1, and Vim1) in the kidneys of WT and TLR7 KO mice fed a chow diet or adenine diet for 3 weeks. #*p* < 0.05 versus chow diet-fed WT mice. *p < 0.05 versus adenine diet-fed WT mice. **F** COL1 and αSMA protein levels were detected in the kidneys of WT and TLR7 KO mice fed a chow diet- or adenine diet for 3 weeks via western blotting. α-tubulin was used as a loading control. **G** Representative images of Sirius red (SR) staining of the kidneys of WT and TLR7 KO mice fed a chow diet or adenine diet for 3 weeks. Scale bar = 50 μm. **H** SR staining positive areas were calculated to determine the extent of fibrosis in each group. #*p* < 0.05 versus chow diet-fed WT mice. *p < 0.05 versus adenine diet-fed WT mice. **I** Representative ISH images of Col1a1 (Green) gene in the kidneys of WT and TLR7 KO mice fed chow diet or adenine diet. Scale bar = 50 μm

To determine whether increased TLR7 expression contributes to kidney inflammation and fibrosis, TLR7 KO mice and their littermates were fed AD for 3 weeks (Fig. 2A). BUN levels were lower in TLR7 KO mice than in WT mice (Fig. 2B). The protein level of KIM-1 was also lower in TLR7 KO mice than in WT mice (Fig. 2C). Structural changes in the kidneys were determined using H&E staining. Compared with WT mice, TLR7 KO mice showed less tubule dilation (Fig. 2D). We proceeded to examine the extent of fibrosis in a mouse model. TLR7 deficiency resulted in a significantly lower expression of fibrosis-related genes (*Tgfb1, Acta2, Col1a2, Col3a1,* and *Vim)* in TLR7 KO mice than in WT mice (Fig. 2E). The protein levels of collagen I and α-SMA displayed a similar tendency to their gene-level changes (Fig. 2F). The extent of renal fibrosis was confirmed by SR staining. TLR7-deficient kidneys had fewer fibrotic regions than WT kidneys (Fig. 2G, H). Increased *Col1a1* expression was confirmed by ISH. TLR7 KO kidneys had lower *Col1a1* expression than WT kidneys (Fig. 2I). These results suggest that TLR7 deficiency reduced AD-induced renal damage and fibrosis.

### TLR7 deficiency protects against AD-induced renal inflammation

**Fig. 3:**
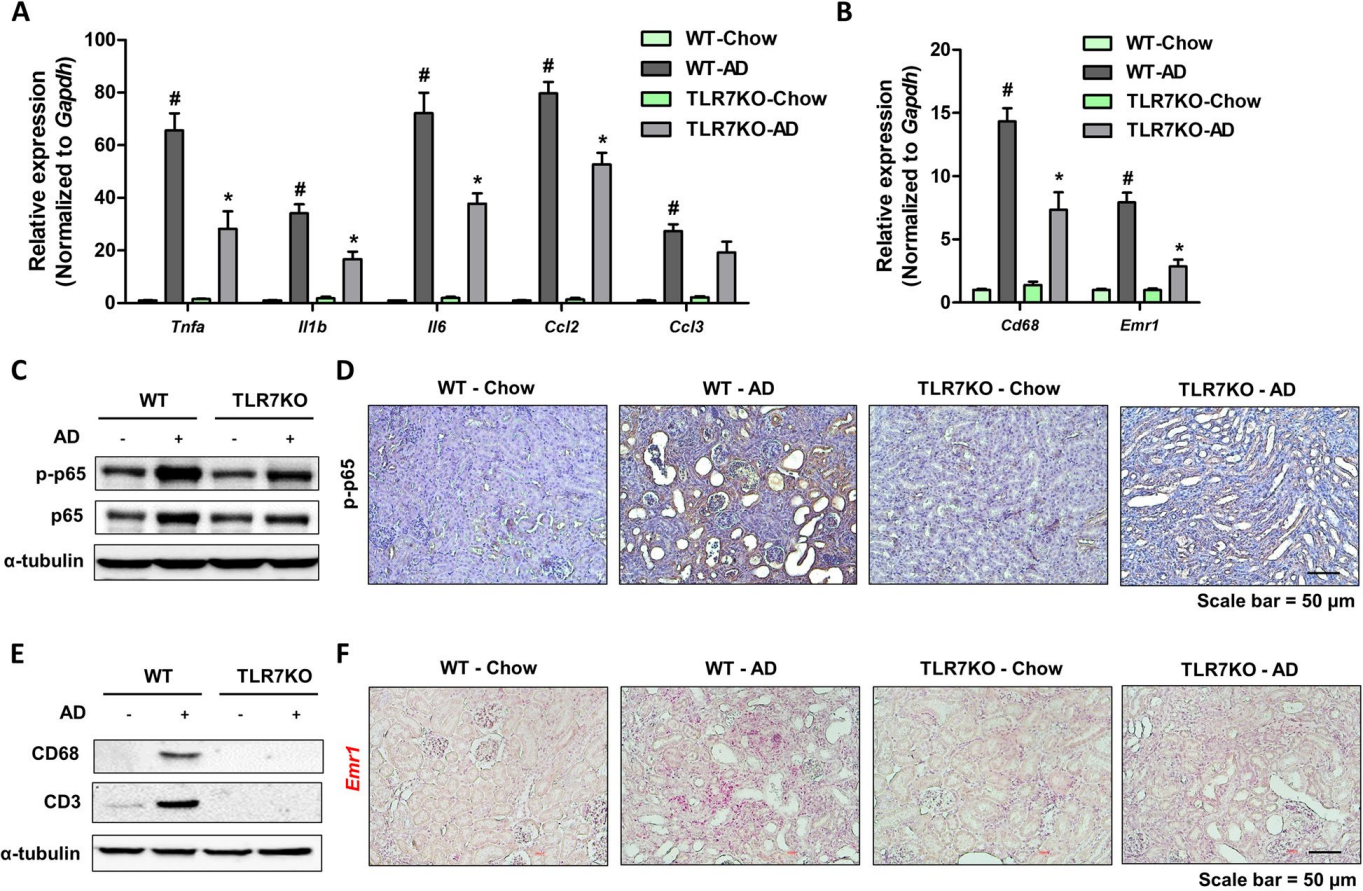
TLR7-deficient kidneys are protected from inflammation. **A** Relative mRNA levels of pro-inflammatory cytokines (Tnfa, Il1b, Il6, Ccl2, and Ccl3) in the kidneys of WT and TLR7 KO mice fed a chow diet or adenine diet for 3 weeks. #*p* < 0.05 versus chow diet-fed WT mice. *p < 0.05 versus adenine diet-fed WT mice. **B** Relative mRNA levels of inflammatory cell marker genes (Cd68 and Emr1) in the kidneys of WT and TLR7 KO mice fed a chow diet or adenine diet for 3 weeks. #*p* < 0.05 versus chow diet-fed WT mice. *p < 0.05 versus adenine diet-fed WT mice. **C** Protein levels of p65 and phosphorylated-p65 in the kidneys were determined. α-tubulin was used as a loading control. **D  **Representative IHC images of phosphotylated-p65 expression in the kidneys. Scale bar = 50 μm. **E** Protein levels of CD68 and CD3 were determined in the kidneys. α-tubulin was used as a loading control. **F** Representative ISH images of Emr1 (Red) gene in the kidneys of WT and TLR7 KO mice fed chow diet or adenine diet. Scale bar = 50 μm

The activation of TLR7 plays an important role in cytokine and chemokine production, thereby increasing inflammatory responses under various pathophysiological conditions. We examined the extent of inflammation in the same mouse model. The gene expression levels of inflammatory cytokines and chemokines (*Tnfa*, *Il1b*, *Il6, Ccl2*, and *Ccl3*) and macrophage markers (*Emr1* and *cd68*) showed less increase in TLR7 deficient kidney relative to those in WT kidneys (Fig. 3A, B). We measured the activation of NF-κB induced by AD under TLR7 deficient conditions. TLR7 deficiency significantly reduced the protein expression of p65 and phosphorylated-p65 (Fig. 3C). Similar to TLR7 expression, p-p65 expression was detected in the damaged tubule epithelial regions (Fig. 3D). However, the expression of immune cell marker proteins was only detected in WT kidneys fed an AD diet (Fig. 3E). The decrease in macrophage infiltration was further confirmed by ISH analysis. Emr1 expression was significantly lower in TLR7 deficient kidney than in WT kidneys (Fig. 3F). Collectively, these data suggest that TLR7 deficient kidneys are protected from AD-induced inflammation.

### TLR7 activation increases chemokine expression in renal tubule epithelial cells

**Fig. 4:**
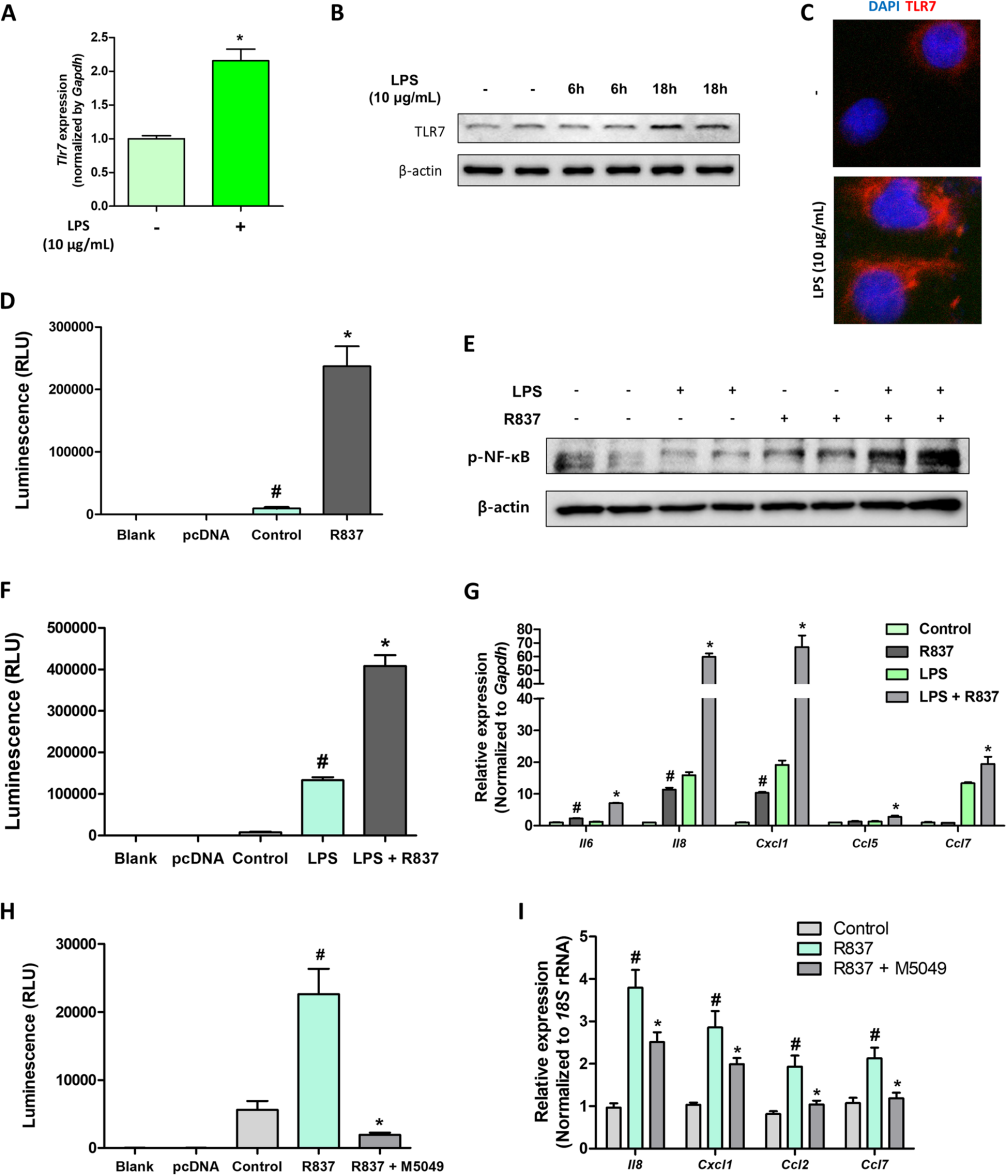
Activation of the TLR7 signaling pathway increases NF-κB activity and chemokine expression in renal tubule epithelial cells. **A** Gene levels of Tlr7 were measured in NRK52E cells treated with lipopolysaccharide (LPS). *p < 0.05 versus non-treated cells. **B** Protein levels of TLR7 were measured in NRK52E cells treated with LPS. β-actin was used as a loading control. **C** Protein expression of TLR7 in NRK52E cells under the LPS-treated condition was detected via immunofluorescence. **D** NF-κB activity in NRK52E cells treated with R837 was measured using the NF-κB promoter luciferase assay. #*p* < 0.05 versus pcDNA group. *p < 0.05 versus control group. **E** Protein levels of phosphorylated-p65 were measured in NRK52E cells treated with LPS or R837. β-actin was used as a loading control. **F** NF-κB activity in NRK52E cells treated with LPS and R837 was measured using the NF-κB promoter luciferase assay. #*p* < 0.05 versus control group. *p < 0.05 versus LPS treated group. **G** Relative mRNA levels of pro-inflammatory chemokines (Il6, Il8, Cxcl1, Ccl5 and Ccl3) in NRK52E cells treated with LPS or R837. #*p* < 0.05 versus control group. *p < 0.05 versus LPS treated group. **H** NF-κB activity in NRK52E cells treated with R837 and M5049 was measured using the NF-κB promoter luciferase assay. #*p* < 0.05 versus control group. *p < 0.05 versus R837-treated group. **I** Relative mRNA levels of pro-inflammatory chemokines (Il8, Cxcl1, Ccl2, and Ccl7) in NRK52E cells treated with R837 and M5049. #*p* < 0.05 versus control group. *p < 0.05 versus LPS treated group

To demonstrate the proinflammatory role of TLR7 in renal epithelial cells, we conducted in vitro experiments using NRK52E rat kidney epithelial cells. First, we examined the expression of TLR7 in epithelial cells. Weak expression of TLR7 was found in epithelial cells. However, inflammatory stimulus by LPS increased TLR7 expression in the cells (Fig. 4A–C). We proceeded to determine whether TLR7 activation increased pro-inflammatory signaling in cells. Treatment with the TLR7 agonist, R837, significantly increased NF-κB activity, as determined via the promoter luciferase assay (Fig. 4D). Phosphorylation of p65 was also increased by R837 treatment, and the extent was higher under LPS-treated conditions (Fig. 4E). The increase in NF-κB activity by R837 treatment was also higher under LPS-treated conditions, suggesting that LPS increased TLR7 expression in the cells (Fig. 4F). When chemokine expression was examined in cells, R837 treatment was found to increase chemokine expression in cells, and R837 treatment under LPS-primed conditions caused a further increase in this expression (Fig. 4G). Finally, we determined whether R837 treatment induced NF-κB activation and chemokine expression via a TLR7-dependent pathway. Pre-treatment with a TLR7 inhibitor (M5049) significantly blocked R837-induced NF-κB activation and chemokine expression in cells (Fig. 4H, I). These findings suggest that TLR7 activation induces NF-κB activation and chemokine expression in renal tubule epithelial cells.

### TLR7 activation by miR-21 induces pro-inflammatory response in epithelial cells

**Fig. 5:**
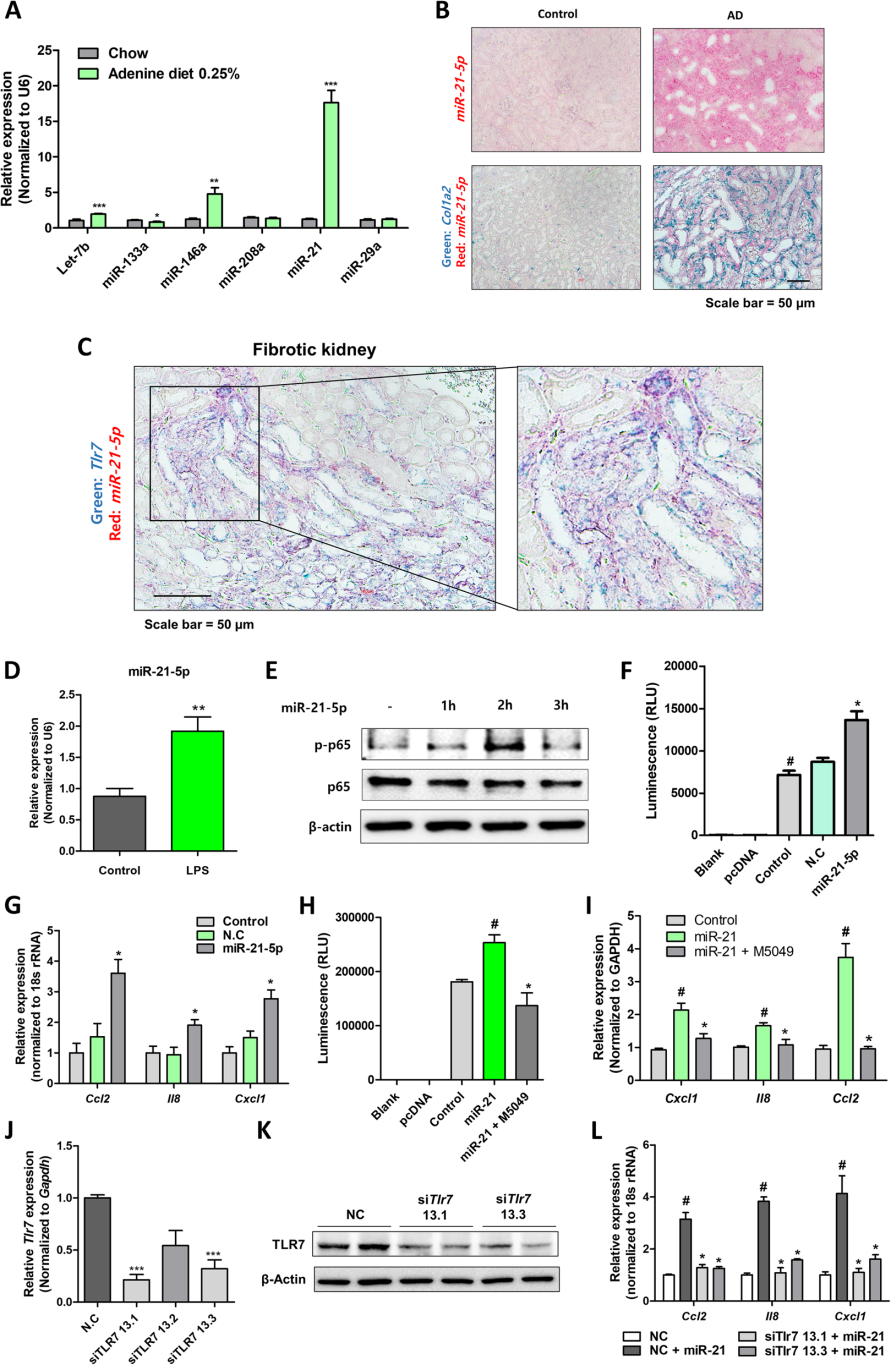
miR-21 induces pro-inflammatory response through TLR7 activation. **A** Relative mRNA levels of miRNAs (Let-7b, miR-133a, miR-148a, miR-208a, miR-21, and miR-29a) in the kidneys of mice fed normal chow diet or adenine diet for 3 weeks. *p < 0.05 versus normal chow diet-fed mice. **p < 0.01 versus normal chow diet-fed mice. ***p < 0.001 versus normal chow diet-fed mice. **B** Representative ISH images of mir-21-5p (red) in the kidneys of mice fed a chow diet or adenine diet (upper panner). Representative ISH images of mir-21-5p (red) and Col1a2 (green) in the kidneys of mice fed a chow diet or adenine diet (lower panner). Scale bar = 50 μm. **C** Representative ISH images of mir-21-5p (red) and Tlr7 (green) in fibrotic kidney. Scale bar = 50 μm. **D** Relative mRNA levels of miR-21-5p in NRK52E cells treated with LPS were measured. **p < 0.01 versus control cells. **E** Protein levels of p65 and phosphorylated-p65 in NRK52E cells transfected with miR-21-5p were measured. β-actin was used as a loading control. **F** NF-κB activity in NRK52E cells treated with miR-21-5p was measured using the NF-κB promoter luciferase assay. #*p* < 0.05 versus blank group. *p < 0.05 versus control group. **G** Relative mRNA levels of pro-inflammatory chemokines (Ccl2, Il8, and Cxcl1) in NRK52E cells treated with miR-21-5p. *p < 0.05 versus N.C treated group. **H** NF-κB activity in NRK52E cells treated with miR-21-5p and M5049 was measured using the NF-κB promoter luciferase assay. #*p* < 0.05 versus control group. *p < 0.05 versus miR-21 treated group. **I** Relative mRNA levels of the pro-inflammatory chemokines (Cxcl1, Il8, and Ccl2) in NRK52E cells treated with miR-21-5p and M5049. #*p* < 0.05 versus control group. *p < 0.05 versus miR-21 treated group. **J** Relative Tlr7 mRNA levels in NRK52E cells transfected with TLR7 siRNA. ***p < 0.001 versus negative control group. **K** Protein level of TLR7 in NRK52E cells transfected with TLR7 siRNA. β-actin was used as a loading control. **L** Relative mRNA levels of the pro-inflammatory chemokines (Cxcl1, Il8, and Ccl2) in NRK52E cells transfected with TLR7 siRNA. miR-21 was further transfected after TLR7 knockdown. #*p* < 0.05 versus negative control group. *p < 0.05 versus negative control and miR-21 transfected group

TLR7 is an innate immune sensor that can detect single-stranded ribonucleic acid. Previous studies have shown that miRNAs are endogenous TLR7 ligands, particularly under sterile inflammatory conditions. Herein, miRNAs previously known as TLR7 ligands were screened. Among the screened miRNAs, *miR-21* expression was significantly increased under fibrotic conditions (Fig. 5A). When the expression region of *miR-21-5p* in the fibrotic kidney was examined, *miR-21-5p* was identified to be highly expressed in the kidney tubule epithelial regions and *miR-21-5p* expression was associated with *Col1a1* expression in the kidney (Fig. 5B). Critically, when the expression of *Tlr7* and *miR-21-5p* were simultaneously detected, the expression regions of the two genes were almost identical (Fig. 5C). To determine whether miR-21-5p acts as an endogenous TLR7 activator in the kidney, in vitro experiments were conducted using NRK52E cells. Under the inflammatory conditions induced by LPS treatment, miR-21-5p expression significantly increased in the cells (Fig. 5D). Transfection with miR-21-5p also increased the phosphorylation of p65 and NF-κB activity in cells, followed by chemokine induction (Fig. 5E ~ G). To demonstrate whether miR-21-5p increases inflammatory responses through TLR7 activation, the TLR7 inhibitor was administered as a pre-treatment before miR-21-5p transfection. M5049 treatment significantly reduced miR-21-5p-induced NF-κB activation and chemokine expression (Fig. 5H and I). We performed additional experiments in which we used a specific small interfering RNA (siRNA) targeting TLR7 to knock down its expression. The TLR7 siRNA was successful in significantly reducing both the RNA and protein levels of TLR7 (Fig. 5J, 5K). Furthermore, we examined the effects of TLR7 silencing on the ability of miR-21 transfection to increase chemokine gene levels. When TLR7 was silenced, the introduction of miR-21 did not lead to an upregulation of chemokine gene levels (Fig. 5L). These data suggest that miR-21 activates NF-κB and induces proinflammatory responses in renal tubule epithelial cells via TLR7 activation.

### TLR7 inhibitor alleviates renal damage and inflammation in FA-induced kidney fibrosis model

**Fig. 6:**
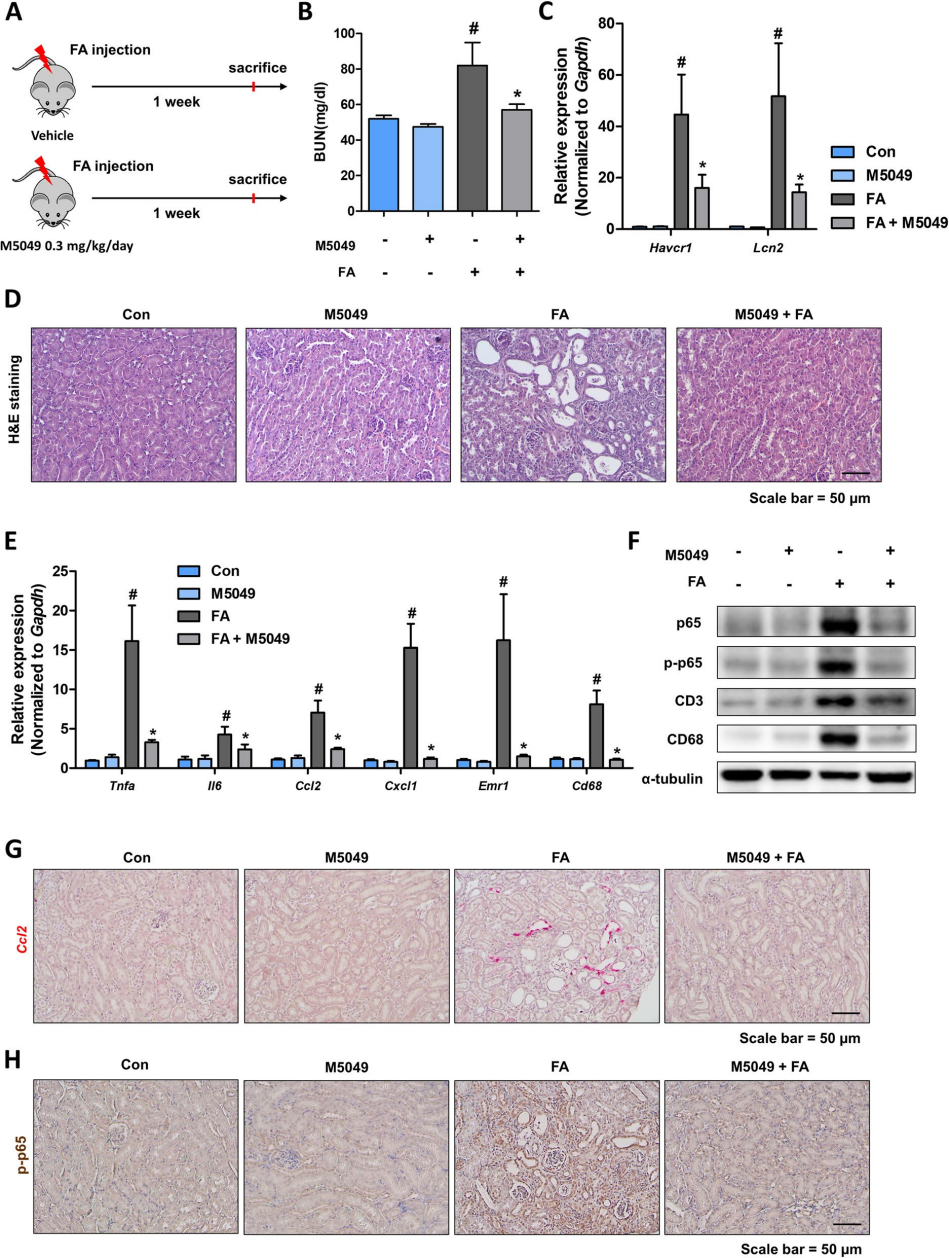
TLR7 inhibition alleviates renal damage and inflammation in the folic acid-treated mouse model. **A** Study design of the administration of M5049 (0.3 mg/kg/day) to the folic acid-induced kidney fibrosis model. **B** Serum BUN levels of folic acid-treated mice with or without M5049 administration. #*p* < 0.05 versus control mice. *p < 0.05 versus folic acid-treated mice. **C** Relative mRNA levels of kidney damage-related genes (Havcr1 and Lcn2) in the kidney of folic acid-treated mice with or without M5049 administration. #*p* < 0.05 versus control mice. *p < 0.05 versus folic acid-treated mice. **D** Representative H&E staining images of folic acid-treated kidneys with or without M5049 administration. Scale bar = 50 μm. **E** Relative mRNA levels of pro-inflammatory cytokines (Tnfa, Il6, Ccl2, and Cxcl1) and inflammatory cell marker genes (Cd68 and Emr1) in the kidney of folic acid-treated mice with or without M5049 administration. #*p* < 0.05 versus control mice. *p < 0.05 versus folic acid-treated mice. **F** Protein levels of p65, p-p65, CD3, and CD68 in the kidneys were determined via western blotting. α-tubulin was used as a loading control. **G** Representative ISH images of Ccl2 (red) in the kidneys. Scale bar = 50 μm. **H** Representative IHC images of p-p65 in the kidneys. Scale bar = 50 μm

**Fig. 7:**
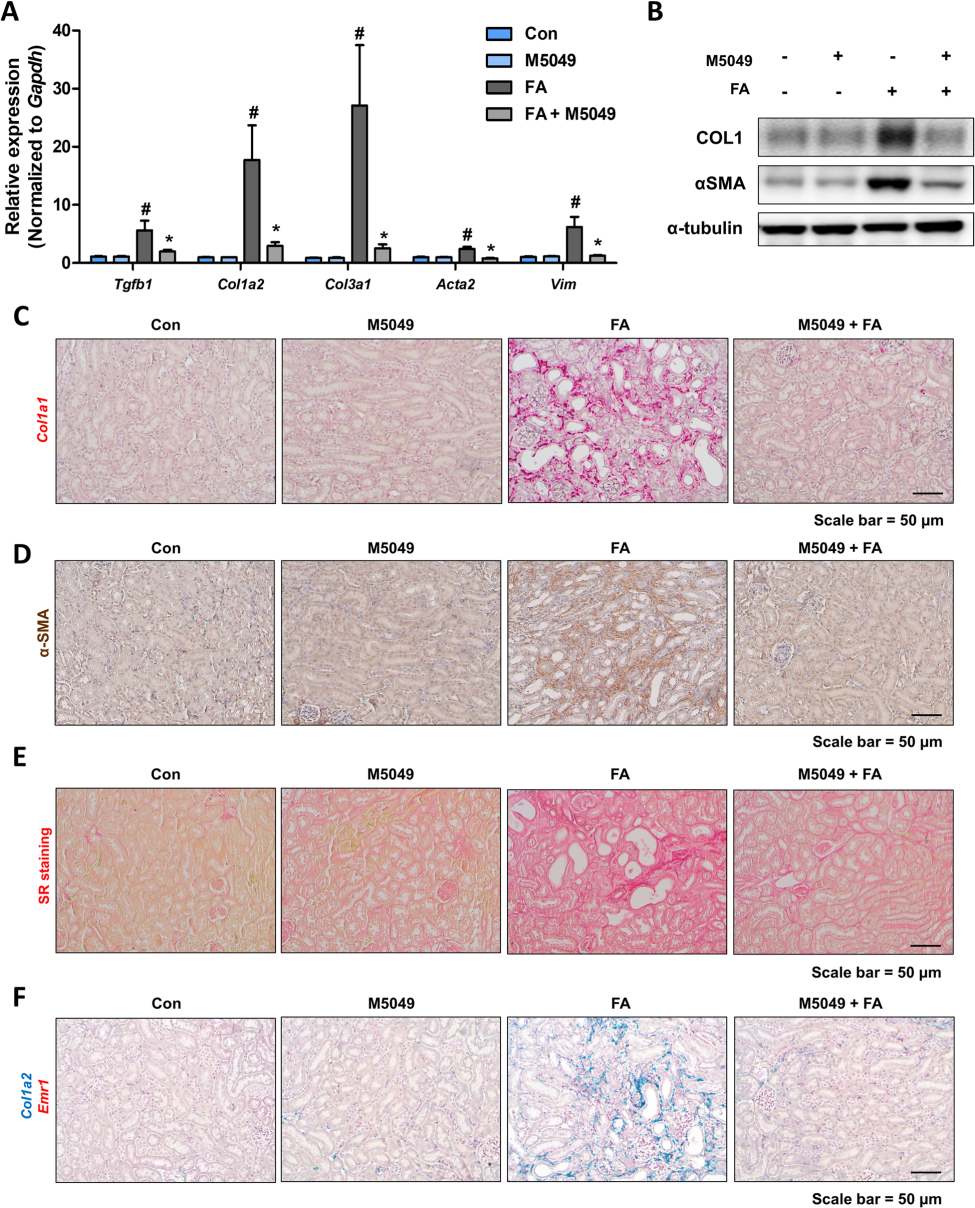
TLR7 inhibition alleviates renal fibrosis in a folic acid-treated mouse model. **A** Relative mRNA levels of fibrosis-related genes (Tgfb1, Col1a2, Col3a1, Acta2, and Vim) in the kidney of folic acid-treated mice with or without M5049 administration. #*p* < 0.05 versus control mice. *p < 0.05 versus folic acid-treated mice. **B** Protein levels of COL1 and αSMA in the kidneys were determined via western blotting. α-tubulin was used as a loading control. **C** Representative ISH images of Col1a1 (red) in the kidneys. Scale bar = 50 μm. **D** Representative IHC images of α-SMA in the kidneys. Scale bar = 50 μm. **E** Representative images of Sirius red (SR) staining in the kidney of folic acid-treated mice with or without M5049 administration. Scale bar = 50 μm. **F** Representative in situ hybridization (ISH) images of the Col1a1 (Green) and Emr1 (Red) genes in the kidney of folic acid-treated mice with or without M5049 administration. Scale bar = 50 μm

Finally, to determine whether TLR7 inhibition can block renal damage and inflammation in kidney fibrosis, in vivo experiments were performed using a FA-induced kidney fibrosis model. The TLR7 inhibitor (M5049, 0.3 mg/kg) was intraperitoneally injected daily during the experimental period (Fig. 6A). TLR7 inhibition significantly reduced BUN levels and the expression levels of kidney damage-related genes (*Havcr1* and *Lcn2*) (Fig. 6B, C). The structural changes in the kidneys, as determined via H&E staining, also included less tubule dilation in the inhibitor-treated group (Fig. 6D). The extent of the inflammation was further examined. The gene expression levels of inflammatory cytokines and chemokines (*Tnfa*, *Il6, Ccl2*, and *Cxcl1*) and macrophage markers (*Emr1* and *cd68*) exhibited a lower increase in TLR7 inhibitor-treated kidneys (Fig. 6E, G). TLR7 inhibition significantly reduced the protein expression of p65 and phosphorylated-p65 (Fig. 6F, H). Thus, we proceeded to examine the extent of fibrosis using the same model. The gene and protein expression of fibrosis markers were significantly reduced in the TLR inhibitor-treated group (Fig. 7A, B). Histological analysis also revealed less fibrotic areas in the TLR7 inhibitor-treated groups (Fig. 7C–E). When the association between inflammation and fibrosis was assessed, *Emr1*- and *Col1a2*-positive cells were found to be localized to the same interstitial region under fibrotic conditions, indicating the importance of inflammation in fibrosis development (Fig. 7F). The TLR7 inhibitor-treated kidneys had fewer *Emr1*- and *Col1a2*-positive regions than FA-treated mice (Fig. 7F). Collectively, these results indicate that TLR7 inhibition alleviates FA-induced renal damage, inflammation, and fibrosis.

## Discussion

In this study, we demonstrated that the activation of TLR7 in renal tubule epithelial cells by miR-21 contributes to inflammation in a renal fibrosis model. The pro-inflammatory role of kidney epithelial cells has been implicated in the development of kidney disease [14]. Damaged tubule cells mainly secrete pro-inflammatory chemokines, which recruit immune cells to evoke inflammation in the kidneys [15, 16]. Blocking pro-inflammatory responses in tubule cells has been demonstrated to have protective effects in animal models of kidney fibrosis [17–19]. Furthermore, by secreting pro-fibrotic growth factors or engaging in the epithelial-mesenchymal transition process, tubule cells significantly contribute to fibrosis development [20]. However, the exact role and mechanism of inflammation initiation in tubular cells are not fully understood. Using a mouse kidney fibrosis model, TLR7 expression was found to be markedly increased in the tubule epithelial regions, and its expression was identified to be associated with macrophage infiltration. Using TLR7 KO mice, we determined the pro-inflammatory role of TLR7. TLR7 deficient kidneys showed reduced inflammation, immune cell infiltration, and fibrosis. Under in vitro conditions, miR-21 acted as an endogenous TLR7 ligand in tubule cells, and TLR7 activation led to increased chemokine expression through NF-κB activation. TLR7 inhibition also reduced kidney damage and inflammation in a fibrosis model. Collectively, our data suggest that the activation of TLR7 by endogenous miR-21 in renal epithelial cells contributes to the inflammatory responses in a renal fibrosis model, suggesting a possible therapeutic target for the treatment of renal fibrosis.

The role of tubular epithelial cells in the initiation of inflammation has been well-demonstrated. In response to stress under damaged conditions, epithelial cells can actively modulate pro-inflammatory responses through the production of cytokines and chemokines [21, 22]. Indeed, under inflammatory conditions during the development of kidney disease, epithelial cells produce several cytokines and chemokines to recruit immune cells [18, 23, 24]. In our study, epithelial cells were also recognized to exhibit proinflammatory features through TLR7 activation. Several previous studies have demonstrated the role of TLR7 in the development of kidney disease. Pawer et al. first described the role of macrophage TLR7 in a model of lupus glomerulonephritis [25]. In the kidney sections of a nephritic mouse model, TLR7 expression was found in infiltrating macrophages and dendritic cells. TLR7 activation increased cytokine and chemokine secretion from cells, and injection of the TLR7 ligand aggravated lupus nephritis in mice [25]. More recently, Zheng et al. described the role of TLR7 in B cells during renal inflammation and Gd-IgA1 synthesis in IgA nephropathy [26]. These researchers found that TLR7 proteins were abundant in CD19^+^ B cells infiltrating the kidneys of patients with IgAN, and inhibition of TLR7 in B cells reduced renal inflammation and nephropathy. Although the pro-inflammatory roles of TLR7 mainly involve immune cells, several recent studies have revealed the possible role of TLR7 in renal tubule cells. Yayi et al. suggested that tubular TLR7 expression is involved in acute kidney ischemia/reperfusion in streptozotocin (STZ)-induced diabetic rats [27]. TLR7 inhibition further attenuates ischemia reperfusion injury in STZ-induced diabetic rat models. In a septic mouse model, TLR7 expression was increased in the kidney tubules [28]. Notably, our results also support the role of TLR7 in kidney tubules. TLR7 expression was almost undetectable in the kidneys under physiological conditions. However, under fibrotic conditions, the expression of TLR7 was highly detectable in dilated tubules. LPS stimulation also increased TLR7 expression in renal tubule epithelial cells in vitro. Tubule epithelial cells stimulated with a TLR7 agonist increased the levels of several well-known chemokines, including Ccl2, Cxcl1, and Il-8. In accordance with the in vitro data, increased Ccl2 expression was detectable in the tubule region of the kidney under fibrotic conditions. Therefore, renal epithelial TLR7 plays an important role in kidney inflammation under disease conditions.

The role of miR-21 has been extensively studied since its discovery. In addition, its role in the development of kidney disease has been reported under various conditions. Chau et al. were the first to report the role of miR-21 in kidney fibrosis [29]. According to these researchers, miR-21 expression was increased in both mouse and human kidney fibrosis. Silencing miR-21 in vivo ameliorates fibrosis and albuminuria in animal models, and the miR-21-mediated silencing of PPARα in epithelial cells plays an important role in metabolic dysregulation during fibrosis. Schauerte et al. described the role of miR-21 in a renal allograft dysfunction model [30]. These researchers found that miR-21, which is mainly secreted by activated macrophages, induces fibroblast activation via Notch2 expression. miR-21 expression was also found to increase in fibrotic kidneys during the screening of miRNAs that were previously known as TLR7 endogenous ligands. miR-21 expression was mainly detected in dilated tubules, and its expression was associated with collagen deposition. Moreover, epithelial cells of dilated tubules that express TLR7 also express miR-21, suggesting a possible relationship between them during kidney fibrosis development. An in vitro analysis was performed to determine whether miR-21 activates TLR7 in renal tubule epithelial cells. The expression of miR-21 was increased under inflammatory conditions. Further, miR-21 treatment significantly increased NF-κB activation and chemokine expression in a TLR7-dependent manner. Based on these observations, we concluded that miR-21 acts as an endogenous ligand of TLR7 and contributes to epithelial inflammation during the development of kidney fibrosis.

Our study aimed to investigate the role of TLR7 in renal inflammation and fibrosis, but there are several limitations. Primarily, our focus was predominantly on understanding the pro-inflammatory role of TLR7 in renal tubule epithelial cells. The adenine diet-induced renal fibrosis model we utilized is known to induce significant inflammation within the kidney, particularly in the renal epithelial and interstitial regions. We observed that TLR7 was mainly expressed in renal tubule epithelial cells, and TLR7 deficient mice exhibited reduced inflammation compared to wildtype mice. This led us to explore the pro-inflammatory roles of TLR7 specifically in renal epithelial cells. We further observed an increase in TLR7 expression following exposure to LPS, providing evidence for the role of TLR7 in inflammation, which is likely contributing to renal fibrosis development. However, the precise mechanisms through which TLR7-mediated inflammation leads to fibrosis remain unclear. In our observations under the fibrotic condition, we noticed an increased infiltration of macrophages near the tubules expressing TLR7. Previous studies have established that infiltrated macrophages play both direct and indirect roles in the development of renal fibrosis [31]. Therefore, it is crucial to conduct further studies to fully understand the specific contribution of TLR7-mediated inflammation to fibrosis development.

## Conclusions

**Fig. 8:**
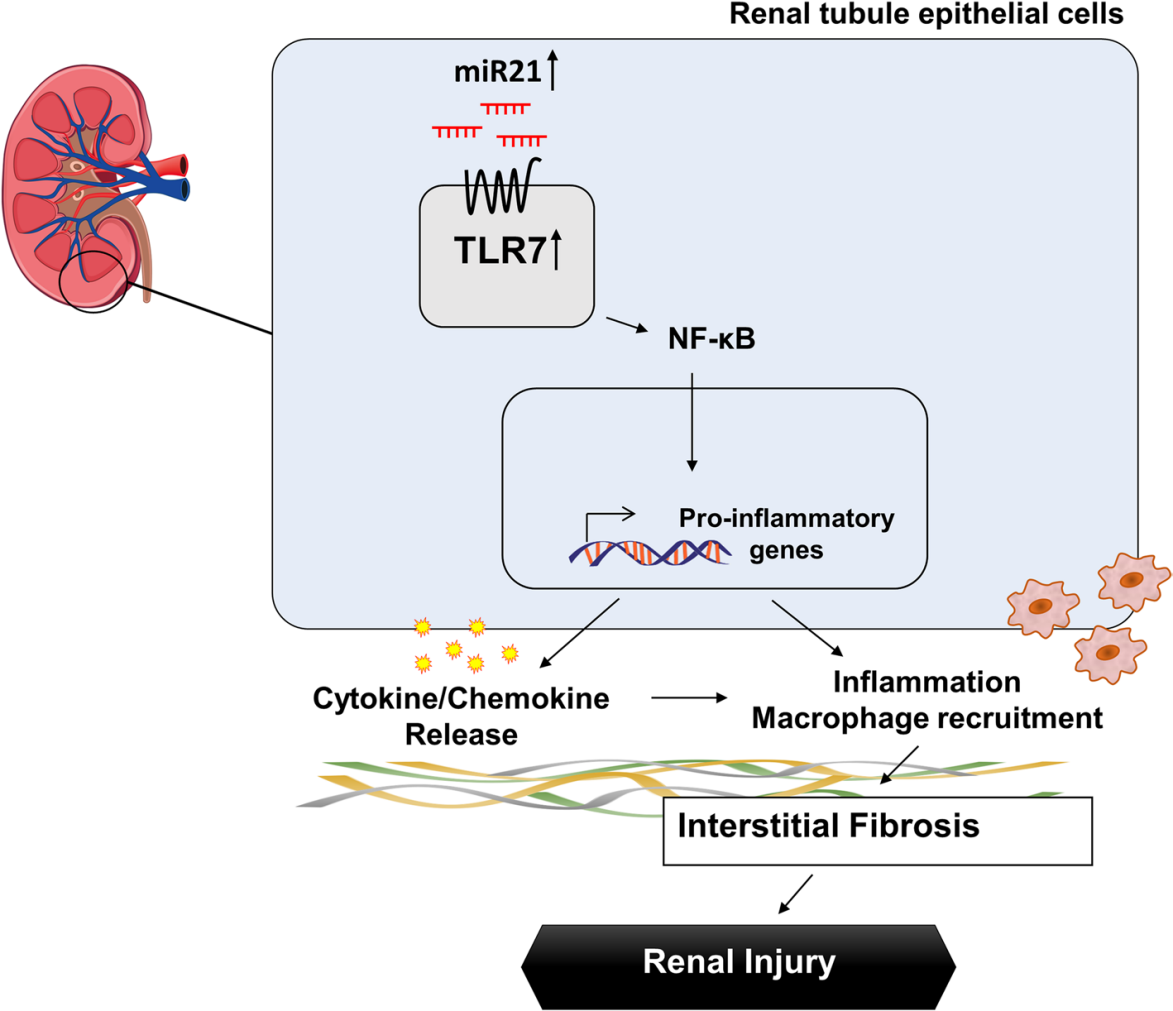
Graphical summary showing the contribution of TLR7 in renal inflammation and fibrosis. TLR7 Activation by miR-21 Promotes Renal Fibrosis by Activating the Pro-Inflammatory Signaling Pathway in Tubule Epithelial Cells

In summary, we determined the role of TLR7 in renal inflammation and fibrosis. Fibrotic kidneys were found to have specifically increased TLR7 levels in the renal tubule epithelial region, with increased inflammatory responses. Using TLR7 deficient mice, epithelial TLR7 was found to play a detrimental role by increasing inflammatory responses. Under in vitro conditions, miR-21-mediated TLR7 activation increased NF-κB activation and chemokine expression. The inhibition of TLR7 by inhibitor treatment also had a protective effect in a mouse model of kidney fibrosis. Together, our data suggest that activation of TLR7 by endogenous miR-21 in renal epithelial cells contributes to the inflammatory responses in a renal fibrosis model, suggesting a possible therapeutic target for the treatment of renal fibrosis (Fig. 8).

### Supplementary Information

**Additional file 1:** **Supplementary Table 1.** Information of Antibodiesused in Western blotting. **SupplementaryTable 2.** Primer sequences for qPCR

## Data Availability

Data sharing is not applicable to this article as no datasets were generated or analyzed during the current study.
