# Supplementary material for: TLR7 activation by miR-21 promotes renal fibrosis by activating the pro-inflammatory signaling pathway in tubule epithelial cells
Source: Cell Commun Signal. 2023 Aug 18;21:215. doi: 10.1186/s12964-023-01234-w (PMC10439664; doi:10.1186/s12964-023-01234-w)
Supplement: Supplementary file 2 — Additional file 1: Supplementary Table 1. Information of Antibodiesused in Western blotting. SupplementaryTable 2. Primer sequences for qPCR [file 12964_2023_1234_MOESM1_ESM.docx]

**Supplementary Table 1. Information of Antibodies used in Western blotting**

| Antibody | Company | Catalog number |
| --- | --- | --- |
| KIM-1 | Abcam | ab 47634 |
| α-SMA | Santa Cruz | sc 32251 |
| p65 | Santa Cruz | sc 514451 |
| p-p65 | Santa Cruz | sc 13548 |
| CD3 | Santa Cruz | sc 20047 |
| CD68 | Santa Cruz | sc 20060 |
| COL1A2 | Santa Cruz | sc 393573 |
| TLR7 | Invitrogen | PA1-28109 |
| TLR2 | Novusbio | NB100-56720 |
| TLR4 | Novusbio | NB100-56723 |
| TLR5 | Novusbio | NBP2-56827 |
| TLR8 | Novusbio | NBP2-24917 |
| ɑ-Tubulin | Santa Cruz | Sc 5286 |

**Supplementary Table 2. Primer sequences for qPCR**

**Mouse**

| *Gene* | *Forward (5’-3’)* | *Reverse (3’-5’)* |
| --- | --- | --- |
| *Col1a2* | *CAGCTCCAGGAAGACCTCGA* | *GTAACAAGGGTGAGCCTGGC* |
| *Col3a1* | *GGTGGCTGCATCCCAATTCA* | *GGCAGGGACAACTGATGGTG* |
| *Vim* | *CAAGCCTGACCTCACTGCTG* | *CACCTGTCTCCGGTACTCGT* |
| *Tgfb1* | *CCTCACCTCCATGTACCAGAA* | *TGGAAATGACCTTGTCAATGAG* |
| *Fn* | *CAACAACCGGAATTACACCG* | *GTCTCGGAGCTGGGAGTAGG* |
| *Acta2* | *TTGCTGACAGGATGCAGAAG* | *TGATCCACATCTGCTGGAAG* |
| *Tnfa* | *CGTCAGCCGATTTGCTATCT* | *CGGACTCCGCAAAGTCTAAG* |
| *Il6* | *AGTTGCCTTCTTGGGACTGA* | *TCCACGATTTCCCAGAGAAC* |
| *Ccl2* | *CCAGCAAGATGATCCCAATG* | *CTTCTTGGGGTCAGCACAGA* |
| *Ccl5* | *CCCTCACCATCATCCTCACT* | *CCTTCGAGTGACAAACACGA* |
| *Cxcl1* | *AATGCATCCACATGCTGCTA* | *ATAGCCTCCTCGACCCACTT* |
| *Emr1* | *TCTGGGGAGCTTACGATGGA* | *GAATCCCGCAATGATGGCAC* |
| *Cd68* | *GGGGCTCTTGGGAACTACAC* | *GTACCGTCACAACCTCCCTG* |
| *Havcr1* | *GTGGAAGTAAAGGGGGTGGT* | *TGCCCCTTTAAGTTGTACCG* |
| *Lcn2* | *CCAGTTCGCCATGGTATTTT* | *GGTGGGGACAGAGAAGATGA* |
| *Tlr1* | *GGACCTACCCTTGCAAACAA* | *GGTGGCACAAGATCACCTTT* |
| *Tlr2* | *TGCTTTCCTGCTGGAGATTT* | *TGTAACGCAACAGCTTCAGG* |
| *Tlr3* | *ATATGCGCTTCAATCCGTTC* | *CGAGAGCATACTGGTGCTGA* |
| *Tlr4* | *GCTTTCACCTCTGCCTTCAC* | *GAAACTGCCATGTTTGAGCA* |
| *Tlr5* | *CTGGGGACCCAGTATGCTAA* | *ACAGCCGAAGTTCCAAGAGA* |
| *Tlr6* | *CAAAGGAGGCGCTATACTCG* | *GGTGGAACAGCCTTGAAAAA* |
| *Tlr7* | *GGAGCTCTGTCCTTGAGTGG* | *CAAGGCATGTCCTAGGTGGT* |
| *Tlr8* | *GGCACAACTCCCTTGTGATT* | *CATTTGGGTGCTGTTGTTTG* |
| *Tlr9* | *ACTGAGCACCCCTGCTTCTA* | *AGATTAGTCAGCGGCAGGAA* |
| *18s* | *CCTGCGGCTTAATTTGACTC* | *AGAACCATCGCTCCACCAAC* |
| *Gapdh* | *AAGGTCATCCCAGAGCTGAA* | *CTGCTTCACCACCTTCTTGA* |

***Mouse miRNA sequence***

| ***Gene name*** | ***Sequence*** |
| --- | --- |
| *Universal primer* | *CTGTGAATGCTGCGACTACGAT* |
| *miR-21-5p* | *UAGCUUAUCAGACUGAUGUUGA* |
| *Let-7b-5p* | *UGAGGUAGUAGGUUGUGUGGUU* |
| *miR-29a-3p* | *UAGCACCAUCUGAAAUCGGUUA* |
| *miR-133a-3p* | *UUUGGUCCCCUUCAACCAGCUG* |
| *miR-146a-5p* | *UGAGAACUGAAUUCCAUGGGUU* |
| *miR-208a-3p* | *ATAAGACGAGCAAAAAGCTTGT* |
| *U6* | *CGCAAGGATGACACGCAAATTC* |

***Rat***

| *Gene* | *Forward (5’-3’)* | *Reverse (3’-5’)* |
| --- | --- | --- |
| *Ccl2* | *GCCAACTCTCACTG AGCCA* | *GCATCTGGCTGAGACAGCAC* |
| *Ccl5* | *ATATGGCTCGGACACCACTC* | *CCACTTCTTCTCTGGGTTGG* |
| *Ccl7* | *AACCAGATGGGACCAATTCA* | *AGATGTGGAACTGGCAGAGG* |
| *Cxcl1* | *GCTGGGATTCACCTCAAGAA* | *TGGGGACACCTTTTAGCATC* |
| *Il6* | *CCGGAGAGGAGACTTCACAG* | *ACAGTGCATCATCGCTGTTC* |
| *Il8* | *GAAGATAGATTGCACCGA* | *CATAGCCTCTCACACATTTC* |
| *Tnfa* | *AGATGTGGAACTGGCAGAGG* | *CCCATTTGGGAACTTCTCCT* |
| *Tgfb* | *ATACGCCTGAGTGGCTGTCT* | *TGGGACTGATCCCATTGATT* |
| *Tlr7* | *GTTTTACGTCTACACAGTAACTCTCTTCA* | *TTCCTGGAGGTTGCTCATGTTTT* |
| *18s* | *AGTCGGCATCGTTTATGGTC* | *CGCGGTTCTATTTTGTTGGT* |
| *Gapdh* | *ACAGCTGCTGCTTTCACCGT* | *TCAACCCACTTCTGATGGGCT* |
